# Association of Time in Range and Time in Tight Range With Cardiovascular Risk Factor Exposure in Youth With Type 1 Diabetes: A Cross‐Sectional Analysis on LDL Cholesterol and Blood Pressure Data From the SWEET International Database

**DOI:** 10.1111/dom.70979

**Published:** 2026-06-15

**Authors:** Claudia Piona, Agata Chobot, Jantje Weiskorn, Arun Shankar, Ingrida Stankute, Katrin Nagl, Marie‐Anne Burckhardt, Christina Kanaka‐Gantenbein, Francesco Gasparini, Marina Cavalin, Eva Hauser, Stefanie Lanzinger, Claudio Maffeis, SWEET Study Group

**Affiliations:** ^1^ Section of Pediatric Diabetes and Metabolism, Department of Surgery, Dentistry, Pediatrics, and Gynecology University of Verona Verona Italy; ^2^ Department of Pediatrics, Institute of Medical Sciences University of Opole Opole Poland; ^3^ Diabetes Centre for Children and Adolescents, Children's Hospital AUF DER BULT Hannover Germany; ^4^ Jothydev's Diabetes Research Center, JDC Junction, Mudavanmugal Trivandrum Kerala India; ^5^ Institute of Endocrinology, Medical Academy Lithuanian University of Health Sciences Kaunas Lithuania; ^6^ Department of Pediatric and Adolescent Medicine Medical University of Vienna Vienna Austria; ^7^ Pediatric Endocrinology and Diabetology University Children's Hospital Basel Basel Switzerland; ^8^ Division of Endocrinology, Metabolism and Diabetes, Diabetes Center and Aghia Sophia Children's Hospital Endo‐ERN Center for Rare Pediatric Endocrine Disorders, First Department of Pediatrics, Medical School National and Kapodistrian University of Athens, “Aghia Sophia” Children's Hospital Athens Greece; ^9^ Department of Pediatrics Women's and Children's Health, Azienda Ospedaliero‐Universitaria Delle Marche, Salesi Hospital Ancona Italy; ^10^ Centro De Diabetes Curitiba Curitiba Paraná Brazil; ^11^ Institute of Epidemiology and Medical Biometry Ulm University Ulm Germany

**Keywords:** blood pressure, cardiovascular risk factors, children, continuous glucose monitoring, LDL‐cholesterol, paediatric, time in range, time in tight range, type 1 diabetes

## Abstract

**Aims:**

To test the hypothesis that continuous glucose monitoring (CGM) metrics are independently associated with cardiovascular risk factor (CVRF) exposure, particularly low‐density lipoprotein cholesterol (LDL‐C) and blood pressure (BP), in youth with type 1 diabetes (T1D) enrolled in the international SWEET registry.

**Materials and Methods:**

This cross‐sectional study included youth aged 6–18 years with T1D duration ≥ 1 year, not receiving antihypertensive or lipid‐lowering therapy and with available CGM profiles (≥ 14 days), lipid and BP data between January 2019 and June 2023. Associations between CGM metrics and LDL‐C or BP were analysed using multiple linear regression, adjusted for sex and pubertal status (model 1) and additionally for body mass index–SDS (model 2), diabetes duration (model 3) and HbA1c (model 4).

**Results:**

Data from 3328 subjects (median age 14.3 years, T1D duration 5.6 years, 52.2% male) were analysed. Time in range (TIR) and time in tight range (TITR) were inversely associated with BP‐SDS and LDL‐C, while time above range (TAR), mean glucose, glucose variability (coefficient of variation (CV)) [%] and glycemia risk index showed positive associations (all *p* < 0.01). In multivariable regression models 1–3, TIR, TITR, TAR and glycemia risk index remained independently associated with LDL‐C and systolic BP‐SDS (*p* < 0.001), with CV also independently associated with systolic BP‐SDS (*p* < 0.001). In model 4 associations with SBP‐SDS were attenuated, whereas those with LDL‐C remained significant, although with a reversal direction.

**Conclusions:**

Key CGM metrics, particularly TIR and TITR, are independently associated with LDL‐C and BP in youth with T1D, highlighting their potential clinical relevance in the assessment of cardiovascular risk profile, alongside HbA1c.

**Abbreviations**
BMI: body mass index
BP: blood pressure
CGM: continuous glucose monitoring
CV: coefficient of variation
CVR: cardiovascular risk
CVRFs: cardiovascular risk factors
DBP: diastolic blood pressure
GRI: glycemia risk index
HDL‐C: high‐density lipoprotein‐cholesterol
LDL‐C: low‐density lipoprotein‐cholesterol
SBP: systolic blood pressure
SDS: standard deviation score
T1D: type 1 diabetes
TAR: time above range
TBR: time below range
TIR: time in range
TITR: time in tight range

## 1 Introduction

In the last decade, the increasing use of Continuous Glucose Monitoring (CGM) led to the definition of standardised CGM‐derived metrics and their clinical targets for assessing glycemic control and complementing information from HbA1c assessment in people living with Type 1 Diabetes (T1D) [1]. Times in different glucose target ranges, particularly Time in Range 70–180 mg/dL (3.9–10.0 mmol/L) (TIR), are recognised as primary CGM outcome measures for both clinical and research settings [2]. Other emerging CGM metrics, including time in tight range 70–140 mg/dL (3.9–7.8 mmol/L) (TITR) and glycemia risk index (GRI), provide additional information on the time spent in ‘normoglycemia’ and the quality of glycaemic control, respectively [3, 4, 5].

A few studies have investigated the association between CGM metrics, focusing mainly on TIR and long‐term diabetes complications, which are the leading cause of morbidity and mortality in adults with T1D [6, 7]. Emerging evidence from these studies suggests that reduced TIR is associated with a higher prevalence of long‐term microvascular and macrovascular complications [6, 7]. Moreover, more recent studies evaluated both TIR and TITR, showing an inverse association between these metrics and the presence of microvascular complications and cerebrovascular events in adults with T1D [8]. Although overt diabetes complications are uncommon in youth with T1D, early subclinical signs of both micro‐ and macrovascular complications can be detected within the first decade after T1D onset and are strongly associated with exposure to cardiovascular risk factors (CVRFs) [9, 10, 11]. Dyslipidaemia and high blood pressure (BP) are considered two major CVRFs, as their early detection and management can prevent the progression of pathogenetic processes that begin in childhood and lead to the development of diabetes complications [9]. Indeed, recently published data from youth with T1D participating in the SWEET registry showed a prevalence of elevated low‐density lipoprotein‐cholesterol (LDL‐C) > 100 mg/dL and high BP of 35% and 17%, respectively [12], confirming previous SWEET reports [13, 14].

A significant association between HbA1c and CVRFs exposure has been consistently demonstrated in the SWEET registry, reinforcing current guideline recommendations to achieve the consensus HbA1c target of ≤ 6.5% (48 mmol/mol), when safely supported by appropriate diabetes technologies [15, 16]. Moreover, although HbA1c was shown to be the strongest modifiable risk factor for cardiovascular diseases in T1D, a substantial proportion of its association with long‐term cardiovascular outcomes seems to be mediated by lipids and systolic blood pressure (SBP) [17].

Nevertheless, data on the relationship between CGM metrics and CVRFs exposure in the paediatric population are limited [7]. A recent cross‐sectional study conducted in Italian children and adolescents with T1D demonstrated that key CGM metrics—TIR, Time Below Range (TBR), Time Above Range (TAR) and coefficient of variation (CV)—were associated with overweight risk in females and with elevated LDL‐C in both females and males, independently of age, diabetes duration, HbA1c, CGM device and insulin therapy modality [18].

Therefore, this study was designed to evaluate the association between CGM metrics, LDL‐C and BP in a large international cohort of children and adolescents with T1D from the SWEET registry. While previous SWEET analyses have examined cardiometabolic risk factors and HbA1c, the independent relationship between standardised CGM metrics and major CVRFs has not been comprehensively investigated in this paediatric multinational cohort. Therefore, this study aimed to test the hypothesis that CGM metrics are independently associated with exposure to these two major CVRFs.

## 2 Materials and Methods

The data analysis was based on the SWEET (‘Better control in Pediatric and Adolescent diabete**S**: **W**orking to cr**E**ate C**E**n**T**ers of Reference’, www.sweet‐project.eu) registry [19]. This multinational network of centres providing care for children, adolescents and young adults with diabetes aims to harmonise and optimise diabetes care by establishing standards of care in paediatric diabetes worldwide, thanks to a prospective, multicentre, standardised diabetes registry.

The centres participating in the SWEET registry submit a set of standardised data to the Institute of Epidemiology and Medical Biometry, Ulm University, in Ulm, Germany, biannually, either using the DPV‐for‐SWEET software (https://sweet.zibmt.uni‐ulm.de/software.php), DIAMAX Digital Exchanges, data download of existing registries or local databases. To increase data quality, inconsistent/implausible data are reported for verification or correction after each data upload. All participating centres fulfil current regulatory data protection security and ethics requirements. Currently, the SWEET network includes 159 diabetes centres from 65 countries worldwide.

**FIGURE 1:**
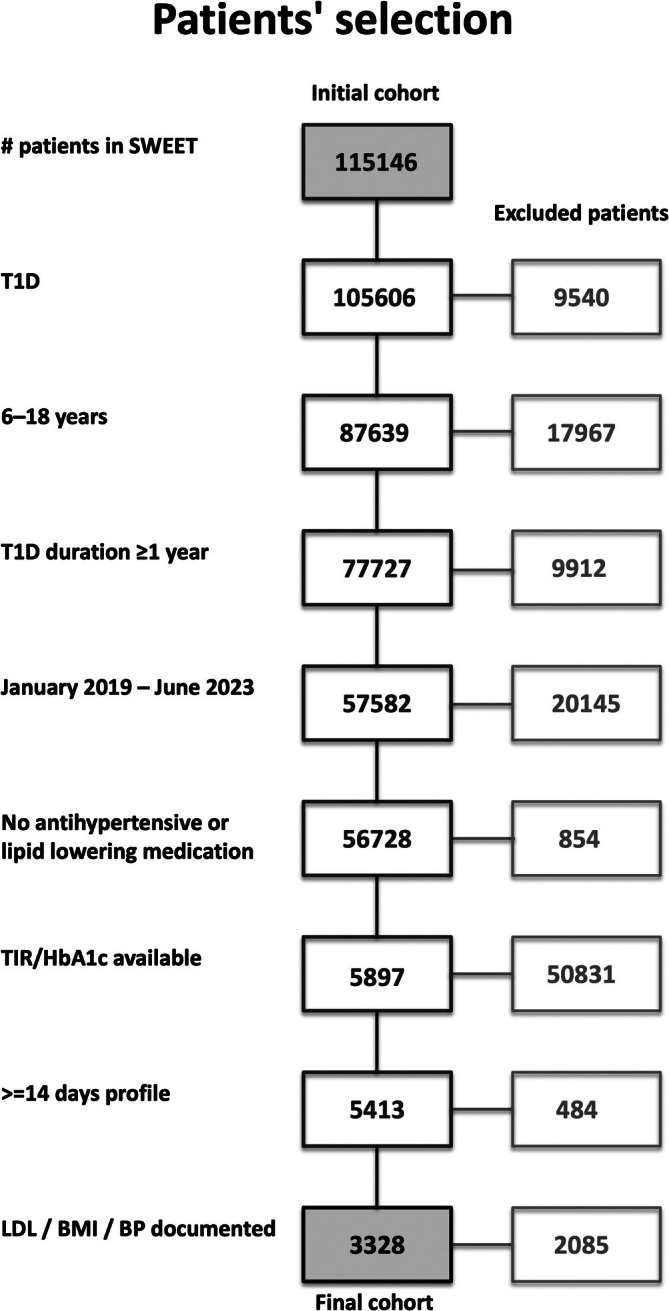
Selection of study population. Flowchart showing the selection of participants included in the analysis. From the overall SWEET database, individuals were excluded if they did not meet the inclusion criteria (age 6–18 years, T1D duration ≥ 1 year), lacked concurrent CGM and cardiovascular risk factor data (LDL‐C and blood pressure), had insufficient CGM data (< 14 days or < 70% completeness) or were receiving lipid‐lowering or antihypertensive treatment.

As of January 2024, the SWEET database contained information on 115 146 participants with diabetes. For the final analysis of this study, children aged 6–18 years with T1D duration ≥ 1 year who had at least 14‐day documented CGM data with > 70% completeness, with available CVRFs data (i.e., LDL‐C, body mass index [BMI] and BP), who were not taking antihypertensive or lipid lowering medication and had available HbA1c data between January 2019 and June 2023 were included (Figure 1). The current analysis involved 13 European countries (26 centres) and five countries (6 centres) outside Europe. For each participant, data were aggregated within the most recent treatment year available during the study period (January 2019 and June 2023). Demographic and clinical parameters, including CGM‐derived metrics, LDL‐C, blood pressure, BMI‐SDS and HbA1c, were summarised using median values within that year, while therapy modality was recorded as the most advanced modality used during the same period. This approach ensured that CGM‐derived metrics and cardiovascular risk factor measurements referred to the same clinical time frame for each individual within the selected treatment year, thereby providing temporal alignment between glycaemic and cardiovascular variables and minimising potential temporal mismatch between these measures. Age, sex, diabetes duration, age at diabetes onset, weight [kg], height [cm], BMI [kg/m^2^], BMI‐SDS, total daily insulin dose per kg bodyweight, HbA1c, CGM metrics, lipid profile and BP [mmHg] and BP‐SDS data were collected from the SWEET database. BMI‐SDS were calculated according to WHO BMI reference charts [20].

HbA1c was measured locally in each centre and standardised to the reference range of the Diabetes Control and Complications Trial [DCCT, 21–43 mmol/mol (4%–6%)] [21].

CGM‐derived metrics were extracted from the most recent available CGM profile within the participant's most recent treatment year during the study period. In line with international consensus recommendations for CGM interpretation, only CGM datasets comprising at least 14 consecutive days with ≥ 70% data completeness were included [1, 2]. A single qualifying 14‐day period within the treatment year was considered sufficient for inclusion.

The following CGM metrics were retrieved from the available CGM profiles (≥ 14 days) according to international recommendations: TIR 70–180 mg/dL (3.9–10.0 mmol/L), TITR 70–140 mg/dL (3.9–7.8 mmol/L), TBR < 70 mg/dL (< 3.9 mmol/L), TBR 54–69 mg/dL (3.0–3.9 mmol/L) (low glucose or Level 1 hypoglycaemia) (TBR1), TBR < 54 mg/dL (< 3.0 mmol/L) (very low glucose or Level 2 hypoglycaemia) (TBR2), TAR > 180 mg/dL (> 10.1 mmol/L), TAR 181–250 mg/dL (10.1–13.9 mmol/L) (high glucose or Level 1 hyperglycaemia) (TAR1), TAR > 250 mg/dL (> 13.9 mmol/L) (very high glucose or Level 2 hyperglycaemia) (TAR2), mean sensor glucose, Coefficient of variation (CV), SD of mean glucose [1, 2]. Moreover, glycemia risk index and its hypoglycaemia and hyperglycaemia components were calculated [5].

Lipid profiles (total cholesterol [TC], high‐density lipoprotein‐cholesterol [HDL‐C], LDL‐C and triglycerides [TG]) were measured at local reference laboratory.

Sex‐, age‐ and height‐specific SBP and diastolic blood pressure (DBP) SDS were calculated using the paediatric normative references [22].

### 2.1 Statistical Analysis

Descriptive results are shown as medians and interquartile range (1st and 3rd quartiles) for continuous variables and as frequencies with percentages for categorical variables. *p*‐Values were generated with the Wilcoxon Rank Sum Test for continuous variables and the Chi‐Square test for discrete variables.

Unadjusted correlations between CGM metrics and CVRFs were assessed using Spearman correlation coefficients and simple linear regression analysis with one CVRF as the outcome variable and one CGM metric as the predictor, separately for each combination of CVRF and CGM metric.

Multiple linear regression models were applied to evaluate variables associated with SBP‐SDS and LDL‐C. CGM metrics (TIR, TITR, TBR, TAR, GRI, GRI hypoglycaemia component, GRI hyperglycaemia component and CV) were entered one at a time for each model. Sex, pubertal status groups, based on age as a surrogate of pubertal status (i.e., prepubertal: females < 10 years, males < 11 years; pubertal: females 10–14 years, males 11–15 years; post‐pubertal: females > 14 years, males > 15 years) were included as covariates in model 1. Model 2 was additionally adjusted for BMI‐SDS; model 3 was additionally adjusted for BMI‐SDS and diabetes duration; model 4 was additionally adjusted for BMI‐SDS, diabetes duration and HbA1c. All regression analyses were conducted using continuous SBP‐SDS and LDL‐C values as outcome variables.

Based on the results of Model 1, the predicted changes in SBP‐SDS and LDL‐C associated with variations in TIR and TITR and the predicted SBP‐SDS and LDL‐C values according to percentile categories of TIR and TITR were calculated. A two‐sided *p*‐value < 0.05 was considered statistically significant. Statistical analysis was performed using Statistical Analysis Software 9.4 (SAS, SAS Institute Inc., Cary, NC, USA).

## 3 Results

### 3.1 Unadjusted Results

This cross‐sectional study included 3328 children and adolescents with T1D (52.2% males). Table 1 shows the demographic, anthropometric, biochemical features and CGM metrics of the overall study population and by sex. Overall, 34.1% of participants had LDL‐C > 100 mg/dL, while 12.4% and 6.6% had systolic BP above the 90th and 95th percentiles, respectively. Median age at the time of the study, age at T1D onset and its duration, total insulin dose, HbA1c and CGM metrics did not differ by sex, except for slightly higher CV in males. BMI‐SDS, all lipid parameters and SBP‐ and DBP‐SDS were higher in females (all *p* < 0.001).

**TABLE 1:** Clinical, anthropometric, biochemical and CGM characteristics of the study population, overall and by sex.

	Females *n* = 1591	Males *n* = 1737	*p*‐Value	Total sample *n* = 3328
Age (years)	14.3 (11.4–16.9)	14.3 (11.4–17.0)	ns	14.3 (11.4–16.9)
Age at onset (years)	7.4 (4.4–10.3)	7.5 (4.5–10.9)	ns	7.4 (4.4–10.5)
Diabetes duration (years)	5.8 (3.1–8.8)	5.4 (3.0–8.7)	ns	5.6 (3.1–8.7)
BMI (kg/m^2^)	21.2 (18.5–23.9)	20.4 (18.0–22.9)	< 0.001	20.8 (18.2–23.4)
BMI‐SDS	0.65 (0.07–1.26)	0.49 (−0.20–1.23)	< 0.001	0.57 (−0.06–1.25)
SBP (mmHg)	112.0 (105.0–119.0)	114.5 (106.5–122.0)	< 0.001	113.0 (105.0–121.0)
SBP‐SDS	0.30 (−0.30–0.93)	0.16 (−0.38–0.76)	< 0.001	0.23 (−0.35–0.84)
DBP (mmHg)	70.0 (65.0–75.0)	69.5 (64.0–74.0)	ns	70.0 (64.5–74.5)
DBP‐SDS	0.51 (0.10–0.92)	0.39 (−0.02–0.75)	< 0.001	0.44 (0.04–0.83)
TC (mg/dL)	169.0 (149.7–189.0)	157.0 (139.0–176.0)	< 0.001	162.0 (144.0–182.9)
TG (mg/dL)	63.0 (49.6–86.0)	58.5 (44.7–82.0)	< 0.001	61.0 (47.0–84.0)
HDL‐C (mg/dL)	60.7 (52.6–70.0)	57.0 (49.0–67.0)	< 0.001	59.0 (51.0–68.0)
LDL‐C (mg/dL)	95.0 (79.5–112.0)	87.0 (71.8–102.9)	< 0.001	90.9 (75.0–107.5)
Total Insulin (U/kg/day)	0.79 (0.64–0.94)	0.77 (0.62–0.94)	ns	0.78 (0.63–0.94)
HbA1c (%, mmol/mol)	7.35 (6.77–8.08)	7.27 (6.75–7.91)	ns	7.30 (6.77–8.00)
	56.8 (50.4–64.8)	55.9 (50.3–62.9)		56.2 (50.4–63.9)
TIR (%)	61.8 (48.3–73.9)	62.7 (50.0–73.3)	ns	62.4 (49.3–73.6)
TITR (%)	37.5 (27.4–47.9)	38.5 (28.4–49.1)	ns	38.1 (27.7–48.5)
TBR (%)	1.1 (0.0–2.5)	1.2 (0.0–3.1)	ns	1.1 (0.0–2.8)
TBR2 (%)	0.0 (0.0–0.0)	0.0 (0.0–0.0)	ns	0.0 (0.0–0.0)
TBR1 (%)	1.1 (0.00–2.5)	1.2 (0.0–3.1)	ns	1.1 (0.00–2.8)
TAR (%)	33.6 (22.6–46.7)	32.5 (22.1–46.0)	ns	32.9 (22.3–46.3)
TAR1 (%)	24.4 (19.1–29.3)	23.7 (19.2–28.6)	ns	24.0 (19.2–29.0)
TAR2 (%)	7.5 (2.1–16.7)	7.5 (2.1–16.5)	ns	7.5 (2.1–16.5)
Mean glucose (mg/dL, mmol/L)	161.1 (146.0–182.4)	159.8 (145.1–181.2)	ns	160.5 (145.5–181.8)
	8.94 (8.09–10.11)	8.86 (8.05–10.05)		8.90 (8.07–10.08)
GRI	38.5 (24.7–56.1)	38.6 (25.6–55.0)	ns	38.6 (25.3–55.4)
GRI hypoglycaemia component	0.8 (0.0–2.0)	0.9 (0.0–2.5)	ns	0.9 (0.00–2.3)
GRI hyperglycaemia component	20.7 (12.3–31.8)	19.8 (12.2–31.1)	ns	20.3 (12.2–31.3)
CV (%)	32.8 (30.0–36.3)	33.8 (30.4–37.1)	0.001	33.2 (30.2–36.7)
SD of mean glucose (mg/dL, mmol/L)	53.7 (45.5–63.4)	54.6 (45.9–64.3)	ns	54.1 (45.7–63.8)
	2.98 (2.52–3.51)	3.03 (2.54–3.56)		2.99 (2.53–3.54)

Abbreviations: BMI, body mass index; CV, coefficient of variation; DBP, diastolic blood pressure; GRI, glycemia risk index; HDL‐C, high‐density lipoprotein‐cholesterol; LDL‐C, low‐density lipoprotein‐cholesterol; ns, not significant; SBP, systolic blood pressure; SD, standard deviation; TAR, time above range > 180 mg/dL (> 10.1 mmol/L); TAR1, time above range 181–250 mg/dL (10.1–13.9 mmol/L) (high glucose or Level 1 hyperglycaemia); TAR2, time above range > 250 mg/dL (> 13.9 mmol/L) (very high glucose or Level 2 hyperglycaemia); TBR, time below range < 70 mg/dL (< 3.9 mmol/L); TBR1, time below range 54–69 mg/dL (3.0–3.9 mmol/L) (low glucose or Level 1 hypoglycaemia); TBR2, time below range < 54 mg/dL (< 3.0 mmol/L) (very low glucose or Level 2 hypoglycaemia); TC, total cholesterol; TG, triglyceride; TIR, time in range 70–180 mg/dL (3.9–10.0 mmol/L); TITR, time in tight range 70–140 mg/dL (3.9–7.8 mmol/L).

Table 2 shows detailed results of the unadjusted correlation analysis between CGM metrics, BP and lipids. TIR and TITR were inversely associated with systolic and diastolic BP‐SDSs and all pro‐atherogenic lipid parameters (TC, TG, LDL‐C) and positively associated with HDL‐C. Hypoglycaemia metrics, in particular TBR1 and GRI hypoglycaemia component, were inversely associated with DBP‐SDS, TG and LDL‐C and positively associated with HDL‐C. Time spent in hyperglycaemia and mean glucose were positively associated with BP‐SDSs and proatherogenic lipids and inversely associated with HDL‐C. CV and SD of mean glucose were positively associated with BP‐SDS and TG.

**TABLE 2:** Unadjusted associations between CGM‐derived metrics and cardiovascular risk factors (blood pressure and lipid parameters) in the study population.

	SBP	SBP‐SDS	DBP	DBP‐SDS	LDL‐C	TC	TG	HDL‐C
%TIR	−0.173***	−0.105***	−0.220***	−0.159***	−0.055*	−0.059**	−0.152***	0.069***
%TITR	−0.163***	−0.091***	−0.221***	−0.158***	−0.062**	−0.060**	−0.141***	0.077***
%TBR	−0.063**	−0.025	−0.094***	−0.073***	−0.050*	−0.017	−0.068***	0.089***
%TBR2	0.006	0.021	−0.036	−0.028	−0.029	0.001	−0.020	0.068***
%TBR1	−0.063**	−0.026	−0.094***	−0.074***	−0.050*	−0.018	−0.069***	0.088***
%TAR	0.153***	0.097***	0.209***	0.161***	0.061**	0.060**	0.154***	−0.080***
%TAR1	0.102***	0.058**	0.150***	0.113***	0.014	0.002	0.060**	−0.080***
%TAR2	0.148***	0.106***	0.193***	0.155***	0.059**	0.068***	0.174***	−0.059**
Mean glucose (mg/dL)	0.152***	0.098***	0.210***	0.163***	0.070***	0.065**	0.161***	−0.085***
GRI	0.165***	0.109***	0.204***	0.154***	0.052*	0.064**	0.157***	−0.048*
GRI hypoglycaemia component	−0.063**	−0.025	−0.095***	−0.073***	−0.050*	−0.017	−0.069***	0.089***
GRI hyperglycaemia component	0.155***	0.102***	0.208***	0.161***	0.062**	0.064**	0.164***	−0.074***
%CV	0.073***	0.069***	0.053*	0.041	−0.005	0.029	0.095***	0.028
SD of mean glucose (mg/dL)	0.148***	0.109***	0.172***	0.133***	0.043	0.061**	0.166***	−0.038

Abbreviations: CV, coefficient of variation; DBP, diastolic blood pressure; GRI, glycemia risk index; HDL‐C, high‐density lipoprotein‐cholesterol; LDL‐C, low‐density lipoprotein‐cholesterol; SBP, systolic blood pressure; SD, standard deviation; TAR, time above range > 180 mg/dL (> 10.1 mmol/L); TAR1, time above range 181–250 mg/dL (10.1–13.9 mmol/L) (high glucose or Level 1 hyperglycaemia); TAR2, time above range > 250 mg/dL (> 13.9 mmol/L) (very high glucose or Level 2 hyperglycaemia); TBR, time below range < 70 mg/dL (< 3.9 mmol/L); TBR1, time below range 54–69 mg/dL (3.0–3.9 mmol/L) (low glucose or Level 1 hypoglycaemia); TBR2, time below range < 54 mg/dL (< 3.0 mmol/L) (very low glucose or Level 2 hypoglycaemia); TC, total cholesterol; TG, triglyceride; TIR, time in range 70–180 mg/dL (3.9–10.0 mmol/L); TITR, time in tight range 70–140 mg/dL (3.9–7.8 mmol/L).

* *p* < 0.01.

** *p* < 0.001.

*** *p* < 0.0001.

### 3.2 Adjusted Results

The results of linear regression analyses with SBP‐SDS or LDL‐C as the outcome variable are shown in Table 3, including additional details presented in Tables [Link], [Link].

**TABLE 3:** Multiple linear regression models evaluating associations of CGM‐derived metrics with SBP‐SDS and LDL‐C in children and adolescents with type 1 diabetes.

Predictor	SBP‐SDS		LDL‐C	
	Model	B (95% CI)	Model	B (95% CI)
TIR	Model 1	−0.005 (−0.007 to −0.003)***	Model 1	−0.125 (−0.176 to −0.074)***
	Model 2	−0.004 (−0.006 to −0.002)***	Model 2	−0.110 (−0.161 to −0.059)***
	Model 3	−0.004 (−0.006 to −0.002)***	Model 3	−0.111 (−0.162 to −0.059)***
	Model 4	0.000 (−0.003 to 0.002)	Model 4	0.131 (0.060 to 0.201)***
TITR	Model 1	−0.005 (−0.007 to −0.003)***	Model 1	−0.125 (−0.182 to −0.068)***
	Model 2	−0.004 (−0.006 to −0.002)***	Model 2	−0.106 (−0.163 to −0.048)***
	Model 3	−0.003 (−0.006 to −0.001)***	Model 3	−0.106 (−0.164 to −0.049)***
	Model 4	0.001 (−0.002 to 0.003)	Model 4	0.156 (0.080–0.232)***
TBR	Model 1	0.003 (−0.008 to 0.014)	Model 1	−0.271 (−0.575 to 0.034)
	Model 2	0.009 (−0.002 to 0.020)	Model 2	−0.209 (−0.513 to 0.095)
	Model 3	0.008 (−0.003 to 0.019)	Model 3	−0.211 (−0.515 to 0.093)
	Model 4	0.015 (0.004 to 0.026)*	Model 4	0.093 (−0.212 to 0.399)
TAR	Model 1	0.005 (0.003–0.006)***	Model 1	0.125 (0.076–0.175)***
	Model 2	0.003 (0.001–0.005)***	Model 2	0.109 (0.059–0.158)***
	Model 3	0.003 (0.001–0.005)***	Model 3	0.110 (0.060–0.160)***
	Model 4	−0.001 (−0.003 to 0.002)	Model 4	−0.133 (−0.202 to −0.064)***
CV	Model 1	0.007 (0.002–0.012)**	Model 1	0.096 (−0.047 to 0.238)
	Model 2	0.007 (0.002–0.012)**	Model 2	0.095 (−0.049 to 0.239)
	Model 3	0.006 (0.001–0.011)**	Model 3	0.095 (−0.049 to 0.239)
	Model 4	0.003 (−0.002 to 0.008)	Model 4	−0.077 (−0.223 to 0.069)
GRI	Model 1	0.004 (0.003–0.005)***	Model 1	0.101 (0.062–0.140)***
	Model 2	0.003 (0.002–0.005)***	Model 2	0.090 (0.051–0.129)***
	Model 3	0.003 (0.002–0.005)***	Model 3	0.091 (0.052–0.131)***
	Model 4	0.001 (−0.001 to 0.003)	Model 4	−0.078 (−0.131 to −0.026)**
GRI hypoglycaemia component	Model 1	0.005 (−0.008 to 0.018)	Model 1	−0.312 (−0.678 to 0.054)
	Model 2	0.011 (−0.002 to 0.024)	Model 2	−0.239 (−0.604 to 0.125)
	Model 3	0.011 (−0.002 to 0.023)	Model 3	−0.242 (−0.607 to 0.124)
	Model 4	0.018 (0.005–0.031)**	Model 4	0.115 (−0.252 to 0.481)
GRI hyperglycaemia component	Model 1	0.006 (0.004–0.008)***	Model 1	0.169 (0.110–0.229)***
	Model 2	0.004 (0.002–0.006)***	Model 2	0.151 (0.092–0.210)***
	Model 3	0.004 (0.002–0.006)***	Model 3	0.152 (0.093–0.212)***
	Model 4	0.000 (−0.003 to 0.003)	Model 4	−0.132 (−0.217 to −0.048)**

*Note:* Estimates with 95% CI in brackets. Model 1: adjusted for sex and pubertal status groups. Model 2: adjusted for sex and pubertal status groups and BMI‐SDS. Model 3: adjusted for sex and pubertal status groups and diabetes duration. Model 4: adjusted for sex and pubertal status groups, BMI‐SDS, diabetes duration and HbA1c.

Abbreviations: CV, coefficient of variation; GRI, glycemia risk index; LDL‐C, low‐density lipoprotein‐cholesterol; SBP, systolic blood pressure; SDS, standard deviation score; TAR, time above range > 180 mg/dL (> 10.1 mmol/L); TBR, time below range < 70 mg/dL (< 3.9 mmol/L); TIR, time in range 70–180 mg/dL (3.9–10.0 mmol/L); TITR, time in tight range 70–140 mg/dL (3.9–7.8 mmol/L).

* *p* < 0.05.

** *p* < 0.01.

*** *p* < 0.001.

Overall, the results showed that the associations between CGM‐derived metrics and SBP‐SDS or LDL‐C differed according to the metric considered and the degree of covariate adjustment. For SBP‐SDS, TIR, TITR, TAR, CV, GRI and the GRI hyperglycaemia component were significantly associated with the outcome in Models 1–3, whereas TBR and the GRI hypoglycaemia component were not. After additional adjustment for HbA1c in Model 4, these associations were attenuated and lost statistical significance, while TBR and the GRI hypoglycaemia component emerged as significant predictors. A different pattern was observed for LDL‐C: TIR, TITR, TAR, GRI and the GRI hyperglycaemia component remained significantly associated across all four models, although the direction of these associations changed after adjustment for HbA1c in Model 4. By contrast, CV, TBR and the GRI hypoglycaemia component were not significantly associated with LDL‐C in any model.

**FIGURE 2:**
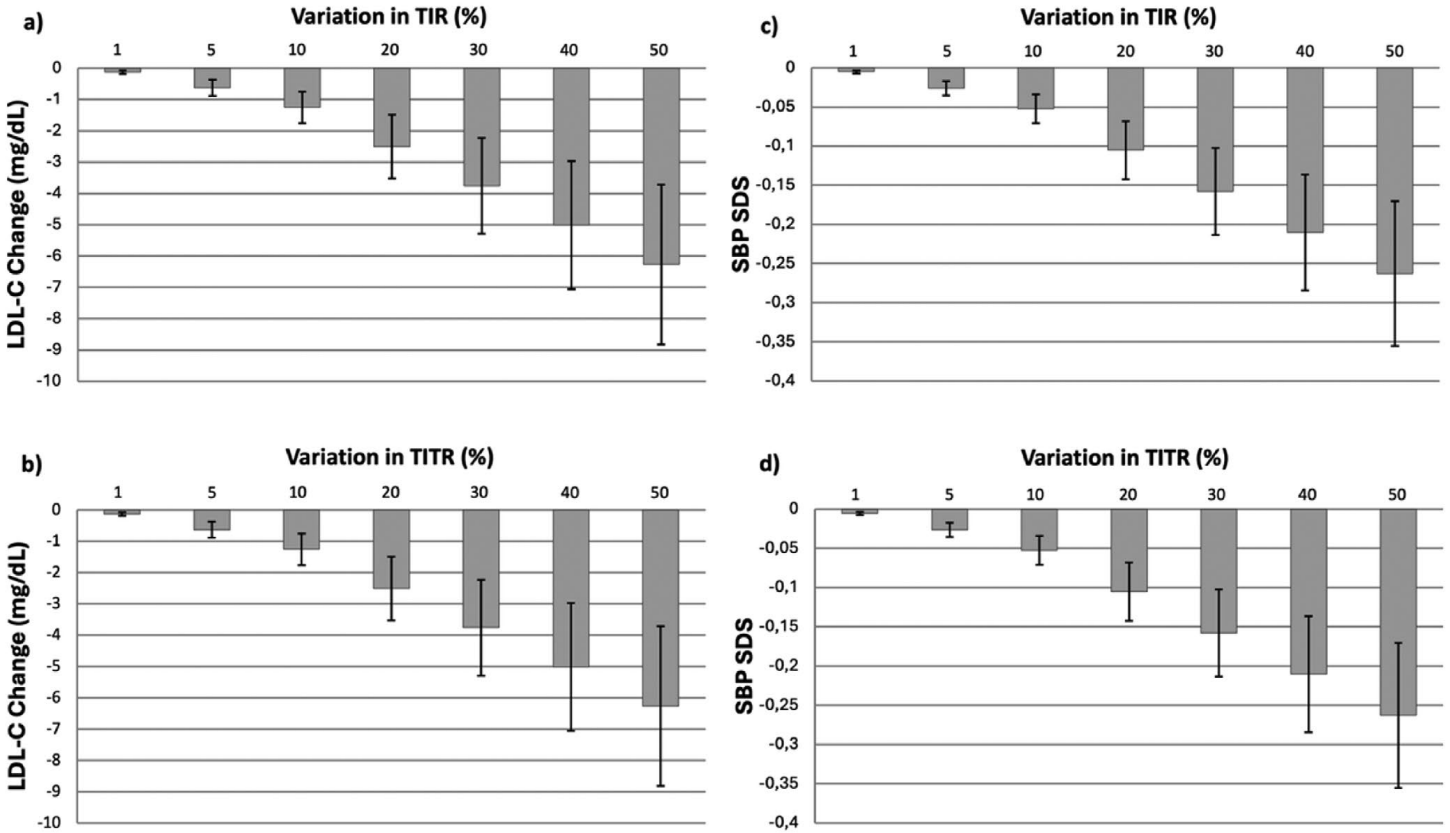
Predicted changes in SBP SDS and LDL‐C associated with variations in TIR and TITR. Legend: TIR, Time in Range 70–180 mg/dL (3.9–10.0 mmol/L); TITR, Time in Tight Range 70–140 mg/dL (3.9–7.8 mmol/L); SBP, systolic blood pressure; LDL‐C, Low‐density lipoprotein‐cholesterol.

Figure 2 illustrates the predicted changes in SBP‐SDS and LDL‐C associated with variations in TIR and TITR, based on model 1. Predicted SBP‐SDS and LDL‐C values according to percentile categories of TIR and TITR are reported in Tables S5 and S6, respectively.

## 4 Discussion

The results of this cross‐sectional study, involving a large international cohort of children and adolescents with T1D, showed that several CGM metrics were independently associated with elevated (systolic) BP and LDL‐C, although these associations varied according to the metric considered, the outcome examined and the degree of model adjustment. This novel finding in the paediatric population highlights the association between key CGM metrics and CVRFs, supporting the complementary use of CGM metrics in addition to HbA1c in the evaluation of glycaemic control and underscoring their potential relevance in the context of long‐term cardiovascular risk assessment.

The association between TIR, a primary CGM outcome and diabetes‐related complications has been increasingly explored in adults with T1D in the last decade. Most evidence has reported an inverse association between higher TIR and CVRF exposure and diabetes complications [6, 7, 8]. Indeed, the odds of a person with diabetes presenting with any microvascular complication or cerebrovascular event decreased, respectively, by 17.2% and 25.1% for each 10% increase in TIR [8]. Moreover, achieving a TIR target > 70% has been associated with a lower prevalence of retinopathy and neuropathy in adults with T1D [6]. Similarly, in children and adolescents with T1D, TIR < 70% was associated with elevated LDL‐C (> 100 mg/dL), independent of sex [18].

In this large, international, paediatric T1D cohort, TIR was independently inversely associated with BP, LDL‐C and other pro‐atherogenic lipid parameters, while showing a positive association with HDL‐C independent of sex and pubertal status group, BMI‐SDS and diabetes duration. After further adjustment for HbA1c, the association with SBP‐SDS was no longer statistically significant, suggesting that part of the observed relationship may be shared with overall glycaemic exposure. For LDL‐C, the association with TIR remained statistically significant even after adjustment for HbA1c; however, its direction changed, indicating a more complex relationship between average glucose exposure, CGM‐derived glucose distribution and lipid metabolism. Given the high prevalence of these CVRFs in children and adolescents with T1D [12] and the evidence supporting the beneficial impact of reducing CVRFs exposure on preventing diabetes complications [9], our findings highlight the association between higher TIR and a more favourable CVRFs profile from the paediatric age, while also indicating that its interpretation should take into account the additional contribution of HbA1c. This aligns with the current HbA1c target recommendation of < 6.5% for individuals with access to advanced diabetes technologies, which corresponds to a TIR of approximately > 80% [15].

Recently, TITR has also been investigated in relation to cardiovascular risk markers and the development of diabetes complications in T1D [8]. De Meulemeester et al. demonstrated, for the first time in adults with T1D, an independent association between TITR and any microvascular complication, diabetic retinopathy and cerebrovascular event [8]. Notably, each 10% increase in TITR was associated with a 34.9% lower incidence of cerebrovascular accident compared to a 25.1% reduction for each 10% increase in TIR. According to this study's results, TITR may provide additional insight beyond TIR in the assessment of CGM profiles in individuals achieving high TIR, with emerging evidence linking this metric to the risk of complications [8, 23]. In this context, our results show that TITR was associated with high blood pressure and unfavourable lipid parameters in the paediatric population after adjustment for sex, pubertal status group, BMI‐SDS and diabetes duration. After further adjustment for HbA1c, the association with SBP‐SDS was no longer statistically significant, whereas the association with LDL‐C remained significant, although its direction changed. However, TITR and TIR showed similar associations with these CVRFs, indicating that our data do not support a clear superiority of TITR over TIR. While evidence from adult populations suggests a potential added value of TITR, our cross‐sectional findings in children and adolescents suggest that both metrics may provide complementary information alongside HbA1c, rather than replacing it, for cardiovascular risk assessment. This interpretation is particularly relevant in light of the attenuation or change in direction of some associations after further adjustment for HbA1c, supporting the view that CGM‐derived metrics and HbA1c capture overlapping but not identical aspects of glycaemic control. Taken together, these metrics may contribute to a more comprehensive and individualised assessment of cardiometabolic risk according to patients' sex and pubertal status.

Longitudinal studies are needed to clarify the respective and complementary prognostic roles of TITR, TIR and HbA1c in paediatric populations with T1D. This is particularly relevant for children and adolescents who, thanks to the use of AID systems, can achieve recommended TIR and TITR targets. In this context, understanding whether TITR adds clinically meaningful information beyond TIR and alongside HbA1c may help refine long‐term cardiovascular risk stratification and prevention of diabetes‐related complications [24, 25].

In this study, the predicted values of SBP‐SDS and LDL‐C according to TIR and TITR percentile values across subgroups defined by sex and pubertal status, as well as the predicted changes in these CVRFs associated with variations in TIR and TITR, were calculated based on models adjusted for sex and pubertal status groups. Differences in the predicted SBP‐SDS and LDL‐C values between the 5th and the 95th percentiles of TIR and TITR were 0.26–0.27 SDS for SBP and 6.2–6.4 mg/dL for LDL‐C across all subgroups. The predicted changes in SBP‐SDS and LDL‐C for each 10% increase in TIR and TITR were approximately −0.05 SDS and −1.25 mg/dL, respectively. These effect sizes are modest and should be interpreted cautiously, as they do not support strong individual‐level prediction. In addition, these estimates are substantially smaller than those typically observed with pharmacological or lifestyle interventions. However, BP and LDL‐C are multifactorial traits influenced by several determinants beyond glycaemic control and therefore relatively small effect sizes are expected in this cross‐sectional registry‐based analysis. Accordingly, our findings might be interpreted as statistically significant but quantitatively limited associations that may be meaningful at the population level and require confirmation in longitudinal studies. Moreover, our results indicate associations across the full range of CGM metrics and cardiovascular risk factors, but do not allow identification of specific TIR or TITR levels associated with clinically abnormal BP or LDL‐C.

Although causal relationships cannot be drawn by the results of this study, the associations observed indicate that higher TIR and TITR levels are linked to more favourable SBP and LDL‐C profiles in the analyses adjusted for sex, pubertal status group, BMI‐SDS and diabetes duration. These findings should be interpreted as reflecting an association rather than a direct effect of CGM metrics on CVRFs. In this context, they may still be relevant for long‐term cardiovascular risk assessment, as cumulative exposure to CVRFs begins early in youth with T1D. Indeed, in the adult general population, lowering SBP by 5 mmHg and LDL‐C by 1 mmol/L (corresponding to around 39 mg/dL) with antihypertensive agents and statins is associated with a roughly 10% and 22% relative risk reduction in major cardiovascular events, respectively, even in the presence of low risk of vascular disease and normal LDL‐C values [26, 27, 28]. Moreover, in individuals with T1D, cumulative exposure to CVRFs, particularly elevated LDL‐C, has been associated with an increased long‐term risk of cardiovascular diseases, including coronary artery disease events, underscoring the importance of early identification and management of CVRFs exposure as early as possible after diabetes onset [29]. Finally, suboptimal glucose control in T1D is linked to a higher prevalence and greater severity of hypertension and dyslipidaemia, supporting the hypothesis that improved glycaemic control may be associated with more favourable blood pressure and lipid profiles, although causality cannot be established in the present cross‐sectional study [9].

Our study also showed that GRI, a novel glycaemic metric designed to reflect overall quality of glycaemic control, as well as hyperglycaemia‐related metrics (TAR and hyperglycaemia component of GRI), were independently associated with SBP and LDL‐C in analyses adjusted for sex, pubertal status group, BMI‐SDS and diabetes duration, whereas for LDL‐C these associations remained evident even after further adjustment for HbA1c, although the direction of association changed after inclusion of HbA1c. These findings, even when referring to novel CGM metrics, align with existing evidence describing associations between hyperglycaemia and adverse cardiovascular alerations, including endothelial dysfunction, increased arterial stiffness and dyslipidaemia and support the relevance of minimising time spent in hyperglycaemia as part of comprehensive glycaemic management. while also indicating that the interpretation of these CGM‐derived metrics should be considered alongside HbA1c [1, 9].

Previous studies reported associations between glycaemic variability, hypoglycaemia and CVRFs in T1D [30]. Increased glycaemic variability has been associated with oxidative stress and endothelial dysfunction, mechanisms implicated in hypertension and dyslipidaemia [7]. Similarly, in adults with T1D, recurrent hypoglycaemia has been linked to arrhythmias and impaired vascular function, which are considered adverse cardiovascular features [31]. In this study, CV, TBR and the GRI hypoglycaemia component were not consistently associated with the outcomes. In particular, no significant association with LDL‐C was observed, whereas for SBP‐SDS the pattern was less consistent, with CV being associated only before adjustment for HbA1c and hypoglycaemia‐related metrics becoming significant only after inclusion of HbA1c. Potential explanations for this discrepancy may include the specific characteristics of our cohort, the use of CGM‐derived metrics based on at least one 14‐day monitoring period within the treatment year and the limited number of hypoglycaemic events captured. Although this approach is consistent with international consensus recommendations for CGM interpretation, it may not fully reflect longer‐term glycaemic variability or less frequent hypoglycaemic exposure. Further studies should explore whether such associations may emerge in specific subgroups, such as pubertal adolescents or individuals with longer diabetes duration and with longer‐term monitoring.

This study focused on the role of CGM metrics as markers associated with CVRF exposure, beyond HbA1c, while explicitly accounting for the impact of HbA1c adjustment on these associations, contributing to the debate on this topic over the last years. Lachin et al., in secondary analyses of the DCCT, reported that the association between TITR, TIR and chronic complications weakened when controlling for HbA1c, reflecting the strong correlations between HbA1c and CGM metrics [32]. This raises the issue of multicollinearity, which complicates the interpretation of models that include both HbA1c and CGM metrics simultaneously, as part of the apparent attenuation may result from overlapping information rather than a lack of distinct association. In our analyses, however, variance inflation factors did not indicate substantial multicollinearity, suggesting that the observed attenuation or reversal of some associations after adjustment for HbA1c is unlikely to be explained solely by statistical collinearity. Rather, these findings support the interpretation that HbA1c and CGM‐derived metrics capture overlapping but not identical aspects of glycaemic control. In particular, HbA1c may primarily reflect mean glycaemic exposure over time, whereas CGM‐derived metrics additionally describe the distribution and patterns of glucose levels, including time spent in hyperglycaemia and glycaemic variability. This may explain why some associations were attenuated, while others changed direction after adjustment for HbA1c. However, more recent analyses suggest that CGM‐derived metrics such as TIR and TITR remain associated with microvascular and macrovascular complications even after adjustment for HbA1c and other risk factors [8]. Altogether, these observations support the complementary clinical value of HbA1c and TIR/TITR as glycaemic metrics, indicating that they should be interpreted together rather than as interchangeable measures, while highlighting the need for longitudinal studies and advanced modelling approaches to clarify their respective association with complication risk.

From a clinical perspective, CGM‐derived metrics such as TIR and TITR may provide complementary information for identifying children and adolescents with a less favourable cardiometabolic profile. In individuals showing persistently suboptimal CGM profiles together with elevated LDL‐C or BP, closer follow‐up and a more comprehensive cardiovascular risk assessment may be considered, including regular evaluation of blood pressure, lipid profile, BMI and optimization of overall diabetes management. Although causality cannot be inferred from our analysis, integrating these metrics into cardiovascular risk assessment may influence clinical decision‐making and healthcare resource allocation. Ensuring equitable access to advanced diabetes technologies could therefore become increasingly important, potentially placing additional demands on health services. At the same time, striving to achieve optimal glycaemic targets may impose psychological pressure on some individuals and families; accordingly, efforts to optimise glycaemic control should be accompanied by appropriate clinical support and a balanced, patient‐centred approach.

The study's main limitation is its cross‐sectional design, which does not allow for causality assessment. We could only explore the associations between CGM metrics and BP and LDL‐C as they were assessed simultaneously and therefore the possibility of reverse causation cannot be excluded. Moreover, individuals with other associated concomitant autoimmune diseases, particularly coeliac disease and autoimmune thyroiditis, were included in our cohort, not allowing us to separately investigate the potential impact of these comorbidities on CVRFs. We acknowledge that the lack of stratified or sensitivity analyses according to these conditions may have influenced the observed associations. CGM metrics were derived from at least one 14‐day period with ≥ 70% data completeness within the treatment year. Although this approach aligns with international recommendations, it may not fully reflect longer‐term glycaemic variability. In addition, the requirement for concurrent availability of CGM and CVRFs data, as well as ≥ 70% CGM completeness, may have introduced selection bias, potentially favouring individuals with greater engagement in diabetes care and access to advanced technologies. Furthermore, the multicentre and multinational nature of the cohort may introduce heterogeneity related to genetic background, differences in clinical practice, healthcare systems and access to diabetes technologies across countries and centres, which could not be fully accounted for in the present analysis. In addition, pubertal status was classified using age‐based categories rather than Tanner staging and should therefore be considered a surrogate measure of pubertal development. Additional limitations include the lack of data on ethnic backgrounds, nutritional habits, health risk behaviours, physical activity, family history of cardiovascular disease and sufficiently standardised information on mode of insulin administration and other potential confounders such as socioeconomic status or markers of insulin resistance in the SWEET registry. These unmeasured factors may have contributed to residual confounding and may partly explain the observed associations, particularly considering that HbA1c is strongly correlated with CGM‐derived metrics and may act as both a confounder and a mediator in their associations with cardiovascular risk factors.

Key strengths of this study arise from the large, international paediatric T1D cohort. We analysed data from more than 3300 children and adolescents across multiple countries worldwide, treated with a broad range of technologies, including insulin pump and real‐time CGM with advanced features (i.e., Low glucose suspend, Predictive Low Glucose Suspend or Hybrid closed loop algorithms). A further strength lies in the completeness of the CGM dataset, which includes all currently recommended CGM metrics, most notably TITR, allowing for a comprehensive assessment of glycaemic control. In addition, the simultaneous availability of CGM‐derived metrics, HbA1c and cardiovascular risk factors enabled a comprehensive evaluation of their interrelationships.

In conclusion, TIR, TITR and other key CGM metrics were independently associated with SBP and LDL‐C, two major CVRFs in children and adolescents with T1D. These findings provide additional insights into the relationship between CGM metrics, particularly TIR and TITR and exposure to these main CVRFs, playing a major role in the development of diabetes complications. Future longitudinal studies are warranted to further evaluate whether these associations persist over time and whether changes in CGM metrics are associated with subsequent differences in CVRFs exposure in youth with T1D.

## Author Contributions

C.P. searched the literature, designed the study, interpreted the data, prepared the figures and wrote the manuscript. C.M. participated in the study design, data interpretation and critically reviewed/edited the manuscript. S.L. and E.H. performed the data analysis, data interpretation and critically reviewed/edited the manuscript. C.P. further elaborated data analysis results. A.C. wrote the manuscript and critically reviewed it. J.W., A.S., I.S., K.N., M.‐A.B., C.K.‐G., F.G. and M.C. critically reviewed and edited the manuscript. All co‐authors approved the final version to be published.

## Funding

The work of SWEET is possible through the support of the following corporate sponsors: Abbott, Boehringer Ingelheim, DexCom Inc., Insulet, Lilly Diabetes Excellent Center, Medtronic Europe, Sanofi.

## Conflicts of Interest

The authors declare no conflicts of interest.

## Supporting information


**Table S1:** Multiple linear regression models evaluating associations between CGM‐derived metrics and SBP‐SDS.
**Table S2:** Multiple linear regression models evaluating associations between CGM‐derived metrics and LDL‐C.

**Table S3:** Multiple linear regression models evaluating associations between CGM‐derived metrics and SBP‐SDS. Model 3: adjusted for sex and pubertal status groups and diabetes duration.; Model 4: adjusted for sex and pubertal status groups, BMI‐SDS, diabetes duration and HbA1c.
**Table S4:** Multiple linear regression models evaluating associations between CGM‐derived metrics and LDL‐C. Model 3: adjusted for sex and pubertal status groups and diabetes duration.; Model 4: adjusted for sex and pubertal status groups, BMI‐SDS, diabetes duration and HbA1c.

**Table S5:** Predicted value of SBP‐SDS and LDL‐C by sex and pubertal groups according to estimated percentile categories of TIR.
**Table S6:** Predicted value of SBP‐SDS and LDL‐C by sex and pubertal groups according to estimated percentile categories of TITR.

## Data Availability

The data that support the findings of this study are available on request from the corresponding author. The data are not publicly available due to privacy or ethical restrictions.
