# Supplementary material for: Association of Time in Range and Time in Tight Range With Cardiovascular Risk Factor Exposure in Youth With Type 1 Diabetes: A Cross‐Sectional Analysis on LDL Cholesterol and Blood Pressure Data From the SWEET International Database
Source: Diabetes Obes Metab. 2026 Jun 15;28(9):7982–92. doi: 10.1111/dom.70979 (PMC13448953; doi:10.1111/dom.70979)
Supplement: Supplementary file 1 [file DOM-28-7982-s001.docx]

Supplementary table 3. Multiple linear regression models evaluating associations between CGM-derived metrics and SBP-SDS. Model 3: adjusted for sex and pubertal status groups and diabetes duration.; Model 4: adjusted for sex and pubertal status groups, BMI-SDS, diabetes duration and HbA1c.

| **Predictor** | **Model 3** | **Model 4** | **Model 3** | **Model 4** | **Model 3** | **Model 4** | **Model 3** | **Model 4** | **Model 3** | **Model 4** | **Model 3** | **Model 4** | **Model 3** | **Model 4** | **Model 3** | **Model 4** |
| --- | --- | --- | --- | --- | --- | --- | --- | --- | --- | --- | --- | --- | --- | --- | --- | --- |
| **Pubertal status groups**  **-puberty**  **-post-puberty** | -0.101 (-0.186 – -0.016)*  -0.067 (-0.152 – 0.019) | -0.107 (-0.192 – -0.022)*  -0.076 (-0.162 – 0.009) | -0.103 (-0.188 – -0.017)*  -0.062 (-0.148 – 0.023) | -0.107 (-0.192 – -0.021)*  -0.075 (-0.160 – 0.011) | -0.092 (-0.177 – -0.006)*  -0.043 (-0.128 – 0.042)) | -0.103 (-0.189 – -0.018)*  -0.073 (-0.159 – 0.012) | -0.101 (-0.186 – -0.015)*  -0.061 (-0.146 – 0.025) | -0.107 (-0.192 – -0.022)*  -0.075 (-0.161 – 0.011) | -0.095 (-0.180 – -0.009)*  -0.043 (-0.129 – 0.042) | -0.102 (-0.187 – -0.017)*  -0.076 (-0.162 – 0.009) | --0.099 (-0.184 – -0.013)*-0.065 (-0.150 – 0.020) | -0.106 (-0.191 – -0.020)*  -0.077 (-0.162 – 0.009) | -0.095 (-0.180 – -0.009)*-0.043 (-0.129 – 0.042) | --0.102 (-0.187 – -0.017)*-0.076 (-0.162 – 0.009) | --0.101 (-0.186 – -0.015)*  -0.063 (-0.148 – 0.023) | -0.107 (-0.192 – -0.022)*  -0.076 (-0.161 – 0.010) |
| **Sex (female)** | 0.103 (0.044 – 0.162)*** | 0.096 (0.038 – 0.155)** | 0.100 (0.041 – 0.159)*** | 0.096 (0.037 – 0.155)** | 0.107 (0.048 – 0.166)*** | 0.099 (0.040 – 0.158)** | 0.102 (0.043 – 0.161)*** | 0.096 (0.037 – 0.154)** | 0.105 (0.045 – 0.164)*** | 0.099 (0.040 – 0.158)** | 0.104 (0.046 – 0.163)*** | 0.098 (0.039 – 0.157)** | 0.105 (0.046 – 0.164)*** | 0.099 (0.040 – 0.158)** | 0.102 (0.044 – 0.161)*** | 0.096 (0.037 – 0.155)** |
| **BMI-SDS** | 0.202 (0.173 – 0.232)*** | 0.200 (0.171 – 0.229)*** | 0.203 (0.174 – 0.232)*** | 0.201 (0.172 – 0.230)*** | 0.209 (0.180 – 0.238)*** | 0.201 (0.172 – 0.230)*** | -0.203 (0.174 – 0.232)*** | 0.201 (0.172 – 0.230)*** | -0.210 (0.181 – 0.240)*** | 0.202 (0.173 – 0.232)*** | -0.203 (0.173 – 0.232)*** | 0.200 (0.171 – 0.229)*** | -0.210 (0.181 – 0.240)*** | 0.202 (0.173 – 0.232)*** | -0.203 (0.173 – 0.232)*** | 0.201 (0.172 – 0.230)*** |
| **Diabetes duration** | 0.003 (-0.005 – 0.012) | 0.004 (-0.005 – 0.012) | 0.004 (-0.004 – 0.013) | 0.004 (-0.005 – 0.012) | 0.004 (-0.005 – 0.012) | 0.003 (-0.006 – 0.011) | 0.004 (-0.004 – 0.013) | 0.004 (-0.005 – 0.012) | 0.005 (-0.003 – 0.014) | 0.003 (-0.006 – 0.011) | 0.003 (-0.005 – 0.012) | 0.003 (-0.005 – 0.012) | 0.005 (-0.003 – 0.014) | 0.003 (-0.006 – 0.011) | 0.004 (-0.005 – 0.012) | 0.004 (-0.005 – 0.012) |
| **HbA1c** | - | 0.074 (0.036 – 0.111)*** | - | 0.084 (0.048 – 0.120)*** | - | 0.074 (0.046 – 0.101)*** |  | 0.087 (0.049 – 0.125)*** |  | 0.085 (0.057 – 0.112)*** |  | 0.065 (0.029 – 0.102)*** |  | 0.085 (0.057 – 0.112)*** |  | 0.081 (0.042 – 0.120)*** |
| **TIR** | -0.004 (-0.006 – -0.002)*** | 0.000 (-0.003 – 0.002) | - | - | - | - | - | - | - | - | - | - | - | - | - | - |
| **TITR** | - | - | -0.003 (-0.006 – -0.001)*** | 0.001 (-0.002 – 0.003) | - | - | - | - | - | - | - | - | - | - | - | - |
| **CV** | - | - | - | - | 0.006 (0.001 – 0.011)** | 0.003 (-0.002 – 0.008) | - | - | - | - | - | - | - | - | - | - |
| **TAR** | - | - | - | - | - | - | 0.003 (0.001 – 0.005)*** | -0.001 (-0.003 – 0.002) | - | - | - | - | - | - | - | - |
| **TBR** | - | - | - | - | - | - | - | - | 0.008 (-0.003 – 0.019) | 0.015 (0.004 – 0.026)* | - | - | - | - | - | - |
| **GRI** | - | - | - | - | - | - | - | - | - | - | 0.003 (0.002 – 0.005)*** | 0.001 (-0.001 – 0.003) | - | - | - | - |
| **GRI hypo** | - | - | - | - | - | - | - | - | - | - | - | - | 0.011 (-0.002 – 0.023) | 0.018 (0.005 – 0.031)** | - | - |
| **GRI hyper** | - | - | - | - | - | - | - | - |  | - |  | - |  | - | 0.004 (0.002 – 0.006)*** | 0.000 (-0.003 – 0.003) |
| Estimates with (95% CI) in brackets. *p<0.05; ** p<0.01; ***p<0.001. Pubertal status reference group: pre-puberty  Abbreviations: SBP, systolic blood pressure; TIR, Time in Range 70–180 mg/dL (3.9–10.0 mmol/L); TITR, Time in Tight Range 70-140 mg/dL (3.9 – 7.8 mmol/L); TBR, Time Below Range <70 mg/dL (< 3.9 mmol/L); TBR1, Time Below Range 54-69 mg/dL (3.0 - 3.9 mmol/L) (low glucose or Level 1 hypoglycaemia); TBR2, Time Below Range <54 mg/dL (<3.0 mmol/L) (very low glucose or Level 2 hypoglycaemia); TAR, Time Above range >180 mg/dL (>10.1 mmol/L); TAR1, Time Above Range 181-250 mg/dL (10.1-13.9 mmol/L) (high glucose or Level 1 hyperglycaemia); TAR2, Time Above Range >250 mg/dL (>13.9 mmol/L) (very high glucose or Level 2 hyperglycaemia); GRI, Glycemia Risk Index; CV, Coefficient of variation; SD, standard deviation. | | | | | | | | | | | | | | | | |

Supplementary table 4. Multiple linear regression models evaluating associations between CGM-derived metrics and LDL-C. Model 3: adjusted for sex and pubertal status groups and diabetes duration.; Model 4: adjusted for sex and pubertal status groups, BMI-SDS, diabetes duration and HbA1c.

| **Predictor** | **Model 3** | **Model 4** | **Model 3** | **Model 4** | **Model 3** | **Model 4** | **Model 3** | **Model 4** | **Model 3** | **Model 4** | **Model 3** | **Model 4** | **Model 3** | **Model 4** | **Model 3** | **Model 4** |
| --- | --- | --- | --- | --- | --- | --- | --- | --- | --- | --- | --- | --- | --- | --- | --- | --- |
| **Pubertal status groups**  **-puberty**  **-post-puberty** | 0.141 (-2.268 – 2.551)-2.459 (-4.879 – -0.038)* | -0.265 (-2.642 – 2.112)-3.153 (-5.544 – -0.762)** | -0.090 (-2.322 – 2.501)  -2.370 (-4.792 – 0.053) | -0.166 (-2.542 – 2.210)-3.116 (-5.506 – -0.726)* | 0.329 (-2.090 – 2.748)  -1.801 (-4.210 – 0.608) | -0.305 (-2.692 – 2.082)-3.423 (-5.818 – -1.027)** | 0.133 (-2.276 – 2.542)  -2.384 (-4.799 – 0.032) | -0.262 (-2.639 – 2.115)-3.259 (-5.647 – -0.870)** | 0.156 (-2.261 – 2.573) -1.862 (-4.271 – 0.547) | 0-0.188 (-2.572 – 2.195)-3.366 (-5.759 – -0.973)** | -0.214 (-2.194 – 2.621)ù-2.403 (-4.817 – 0.012) | -0.320 (-2.700 – 2.060)-3.278 (-5.669 – -0.888)** | 0.162 (-2.255 – 2.579)-1.857 (-4.266 – 0.551) | -0.189 (-2.572 – 2.195)-3.367 (-5.760 – -0.975)** | 0.125 (-2.282 – 2.532)-2.480 (-4.894 – -0.067)* | -0.265 (-2.644 – 2.113)-3.282 (-5.672 – -0.892)** |
| **Sex (female)** | 7.685 (6.022 – 9.348)*** | 7.246 (5.604 – 8.888)*** | 7.616 (5.951 – 9.280)*** | 7.344 (5.703 – 8.984)*** | 7.751 (6.081 – 9.421)*** | 7.297 (5.649 – 8.945)*** | 7.651 (5.988 – 9.313)*** | 7.279 (5.638 – 8.921)*** | 7.634 (5.966 – 9.303)*** | 7.378 (5.732 – 9.023)*** | 7.733 (6.071 – 9.396)*** | 7.230 (5.586 – 8.875)*** | 7.637 (5.968 – 9.305)*** | 7.378 (5.733 – 9.023)*** | 7.677 (6.015 – 9.338)*** | 7.263 (5.620 – 8.907)*** |
| **BMI-SDS** | 2.336 (1.512 – 3.161)*** | 2.194 (1.381 – 3.008)*** | 2.349 (1.522 – 3.175)*** | 2.227 (1.413 – 3.041)*** | -2.526 (1.705 – 3.348)*** | 2.103 (1.289 – 2.918)*** | -2.312 (1.487 – 3.138)*** | 2.220 (1.406 – 3.035)*** | 2.487 (1.664 – 3.311)***- | 2.126 (1.311 – 2.941)*** | 2.349 (1.526 – 3.173)***- | 2.156 (1.342 – 2.969)*** | 2.490 (1.666 – 3.314)*** | 2.126 (1.311 – 2.941)*** | 2.297 (1.473 – 3.121)***- | 2.183 (1.369 – 2.997)*** |
| **Diabetes duration** | -0.040 (-0.281 – 0.200) | -0.033 (-0.270 – 0.204)) | -0.026 (-0.266 – 0.214) | -0.037 (-0.273 – 0.200) | -0.003 (-0.246 – 0.240) | -0.055 (-0.294 – 0.185) | -0.034 (-0.274 – 0.206) | -0.041 (-0.277 – 0.196) | 0.032 (-0.207 – 0.272) | -0.080 (-0.317 – 0.157) | -0.049 (-0.289 – 0.192) | -0.038 (-0.276 – 0.199) | 0.032 (-0.207 – 0.271) | -0.080 (-0.317 – 0.157) | -0.044 (-0.284 – 0.196) | -0.047 (-0.284 – 0.190) |
| **HbA1c** | - | 5.177 (4.136 – 6.217)*** | - | 5.216 (4.208 – 6.224)*** | - | 3.936 (3.161 – 4.711)*** | - | 5.268 (4.210 – 6.326)*** | - | 3.887 (3.118 – 4.656)*** | - | 4.866 (3.845 – 5.887)*** | - | 3.887 (3.118 – 4.655)*** | - | 5.056 (3.972 – 6.139)*** |
| **TIR** | -0.111 (-0.162 – -0.059)*** | 0.131 (0.060 – 0.201)*** | - | - | - | - | - | - | - | - | - |  |  | - | - | - |
| **TITR** | - | - | -0.106 (-0.164 – -0.049)*** | 0.156 (0.080 – 0.232)*** | - | - | - | - | - | - | - |  |  | - | - | - |
| **CV** | - | - | - | - | 0.095 (-0.049 – 0.239) | -0.077 (-0.223 – 0.069) | - | - | - | - | - |  |  | - | - | - |
| **TAR** | - | - | - | - | - | - | 0.110 (0.060 – 0.160)*** | -0.133 (-0.202 – -0.064)*** | - | - | - |  |  | - | - | - |
| **TBR** | - | - | - | - | - | - | - | - | -0.211 (-0.515 – 0.093) | 0.093 (-0.212 – 0.399) | - |  |  | - | - | - |
| **GRI** | - | - | - | - | - | - | - | - | - | - | 0.091 (0.052 – 0.131)*** | -0.078 (-0.131 – -0.026)** |  | - | - | - |
| **GRI hypo** | - | - | - | - | - | - | - | - | - | - | - | - | -0.242 (-0.607 – 0.124) | 0.115 (-0.252 – 0.481) | - | - |
| **GRI hyper** | - | - |  | - | - | - | - | - | - | - | - | - | - |  | 0.152 (0.093 – 0.212)*** | -0.132 (-0.217 – -0.048)** |
| Estimates with (95% CI) in brackets. *p<0.05; ** p<0.01; ***p<0.001. Pubertal status reference group: pre-puberty.  Abbreviations:LDL-C: low-density lipoprotein-cholesterol; TIR, Time in Range 70–180 mg/dL (3.9–10.0 mmol/L); TITR, Time in Tight Range 70-140 mg/dL (3.9 – 7.8 mmol/L); TBR, Time Below Range <70 mg/dL (< 3.9 mmol/L); TBR1, Time Below Range 54-69 mg/dL (3.0 - 3.9 mmol/L) (low glucose or Level 1 hypoglycaemia); TBR2, Time Below Range <54 mg/dL (<3.0 mmol/L) (very low glucose or Level 2 hypoglycaemia); TAR, Time Above range >180 mg/dL (>10.1 mmol/L); TAR1, Time Above Range 181-250 mg/dL (10.1-13.9 mmol/L) (high glucose or Level 1 hyperglycaemia); TAR2, Time Above Range >250 mg/dL (>13.9 mmol/L) (very high glucose or Level 2 hyperglycaemia); GRI, Glycemia Risk Index; CV, Coefficient of variation; SD, standard deviation. | | | | | | | | | | | | | | | | |
