# Supplementary material for: Association of Time in Range and Time in Tight Range With Cardiovascular Risk Factor Exposure in Youth With Type 1 Diabetes: A Cross‐Sectional Analysis on LDL Cholesterol and Blood Pressure Data From the SWEET International Database
Source: Diabetes Obes Metab. 2026 Jun 15;28(9):7982–92. doi: 10.1111/dom.70979 (PMC13448953; doi:10.1111/dom.70979)
Supplement: Supplementary file 2 [file DOM-28-7982-s002.docx]

Supplementary Table 5. Predicted value of SBP-SDS and LDL-C by sex and pubertal groups according to estimated percentile categories of TIR.

| **Predictors** | | | | **Predicted parameters** | |
| --- | --- | --- | --- | --- | --- |
| **Sex** | **Pubertal group** | **TIR (%)** | | **SBP-SDS** | **LDL-C (mg/dl)** |
|  |  | **Percentile** | **Value** |  |  |
| female | pre-puberty | 5th* | 32.7 | 0.55 | 101.4 |
|  |  | 25th | 48.3 | 0.46 | 99.4 |
|  |  | 50th | 61.8 | 0.39 | 97.7 |
|  |  | 75th | 73.9 | 0.33 | 96.2 |
|  |  | 95th* | 88.1 | 0.25 | 94.4 |
| male | pre-puberty | 5th* | 34.3 | 0.40 | 93.1 |
|  |  | 25th | 50.0 | 0.32 | 91.2 |
|  |  | 50th | 62.7 | 0.25 | 89.6 |
|  |  | 75th | 73.3 | 0.20 | 88.3 |
|  |  | 95th* | 88.1 | 0.12 | 86.4 |
| female | puberty | 5th* | 32.7 | 0.44 | 101.4 |
|  |  | 25th | 48.3 | 0.36 | 99.4 |
|  |  | 50th | 61.8 | 0.29 | 97.7 |
|  |  | 75th | 73.9 | 0.22 | 96.2 |
|  |  | 95th* | 88.1 | 0.15 | 94.4 |
| male | puberty | 5th* | 34.3 | 0.30 | 93.1 |
|  |  | 25th | 50.0 | 0.22 | 91.2 |
|  |  | 50th | 62.7 | 0.15 | 89.6 |
|  |  | 75th | 73.3 | 0.10 | 88.2 |
|  |  | 95th* | 88.1 | 0.02 | 86.4 |
| female | post-puberty | 5th* | 32.7 | 0.46 | 98.4 |
|  |  | 25th | 48.3 | 0.38 | 96.5 |
|  |  | 50th | 61.8 | 0.31 | 94.8 |
|  |  | 75th | 73.9 | 0.24 | 93.3 |
|  |  | 95th* | 88.1 | 0.17 | 91.5 |
| male | post-puberty | 5th* | 34.3 | 0.32 | 90.2 |
|  |  | 25th | 50.0 | 0.24 | 88.2 |
|  |  | 50th | 62.7 | 0.17 | 86.6 |
|  |  | 75th | 73.3 | 0.11 | 85.3 |
|  |  | 95th* | 88.1 | 0.04 | 83.4 |
| *Estimates based on the respective group’s (male/female) mean and standard deviation.  Abbreviations: TIR, Time in Range 70-180 mg/dL (3.9 – 10.0 mmol/L); SBP-SDS, systolic blood pressure standard deviation score; LDL-C: low-density lipoprotein-cholesterol. | | | | | |

Supplementary Table 6. Predicted value of SBP-SDS and LDL-C by sex and pubertal groups according to estimated percentile categories of TITR.

| **Predictors** | | | | **Predicted parameters** | |
| --- | --- | --- | --- | --- | --- |
| **Sex** | **Pubertal group** | **TITR (%)** | | **SBP-SDS** | **LDL-C (mg/dl)** |
|  |  | **Percentile** | **Value** |  |  |
| female | pre-puberty | 5th* | 13.5 | 0.52 | 100.9 |
|  |  | 25th | 27.4 | 0.45 | 99.1 |
|  |  | 50th | 37.5 | 0.40 | 97.9 |
|  |  | 75th | 47.9 | 0.34 | 96.6 |
|  |  | 95th* | 61.8 | 0.27 | 94.8 |
| male | pre-puberty | 5th* | 14.5 | 0.39 | 92.8 |
|  |  | 25th | 28.4 | 0.32 | 91.0 |
|  |  | 50th | 38.5 | 0.26 | 89.8 |
|  |  | 75th | 49.1 | 0.21 | 88.5 |
|  |  | 95th* | 63.5 | 0.13 | 86.7 |
| female | puberty | 5th* | 13.5 | 0.42 | 100.8 |
|  |  | 25th | 27.4 | 0.35 | 99.1 |
|  |  | 50th | 37.5 | 0.30 | 97.8 |
|  |  | 75th | 47.9 | 0.24 | 96.5 |
|  |  | 95th* | 61.8 | 0.17 | 94.8 |
| male | puberty | 5th* | 14.5 | 0.28 | 92.7 |
|  |  | 25th | 28.4 | 0.21 | 91.0 |
|  |  | 50th | 38.5 | 0.16 | 89.7 |
|  |  | 75th | 49.1 | 0.10 | 88.4 |
|  |  | 95th* | 63.5 | 0.03 | 86.6 |
| female | post-puberty | 5th* | 13.5 | 0.44 | 98.0 |
|  |  | 25th | 27.4 | 0.37 | 96.3 |
|  |  | 50th | 37.5 | 0.32 | 95.0 |
|  |  | 75th | 47.9 | 0.26 | 93.7 |
|  |  | 95th* | 61.8 | 0.19 | 92.0 |
| male | post-puberty | 5th* | 14.5 | 0.31 | 90.0 |
|  |  | 25th | 28.4 | 0.24 | 88.2 |
|  |  | 50th | 38.5 | 0.18 | 87.0 |
|  |  | 75th | 49.1 | 0.13 | 85.6 |
|  |  | 95th* | 63.5 | 0.05 | 83.8 |
| *Estimates based on the respective group’s (male/female) mean and standard deviation.  Abbreviations: TITR, Time in Tight Range 70-140 mg/dL (3.9 – 7.8 mmol/L); SBP-SDS, systolic blood pressure standard deviation score; LDL-C: low-density lipoprotein-cholesterol. | | | | | |
