# Supplementary material for: Association of Time in Range and Time in Tight Range With Cardiovascular Risk Factor Exposure in Youth With Type 1 Diabetes: A Cross‐Sectional Analysis on LDL Cholesterol and Blood Pressure Data From the SWEET International Database
Source: Diabetes Obes Metab. 2026 Jun 15;28(9):7982–92. doi: 10.1111/dom.70979 (PMC13448953; doi:10.1111/dom.70979)
Supplement: Supplementary file 3 [file DOM-28-7982-s003.docx]

Supplementary table 1. Multiple linear regression models evaluating associations between CGM-derived metrics and SBP-SDS. Model 1: adjusted for sex and pubertal status groups; Model 2: adjusted for sex and pubertal status groups, and BMI-SDS.

| **Predictor** | **Model 1** | **Model 2** | **Model 1** | **Model 2** | **Model 1** | **Model 2** | **Model 1** | **Model 2** | **Model 1** | **Model 2** | **Model 1** | **Model 2** | **Model 1** | **Model 2** | **Model 1** | **Model 2** |
| --- | --- | --- | --- | --- | --- | --- | --- | --- | --- | --- | --- | --- | --- | --- | --- | --- |
| **Pubertal status groups**  **-puberty**  **-post-puberty** | -0.104 (-0.190 – -0.017)*  -0.085 (-0.167 – -0.003)* | -0.095 (-0.180 – -0.011)*- -0.054 (-0.135 – 0.026) | --0.105 (-0.191 – -0.019)*  -0.080 (-0.163 – 0.003) | -0.096 (-0.181 – -0.012)*  -0.048 (-0.128 – 0.032) | --0.091 (-0.178 – -0.004)*  -0.050 (-0.131 – 0.031) | -0.085 (-0.169 – -0.001)*  -0.029 (-0.108 – 0.050) | -0.103 (-0.189 – -0.018)*  -0.077 (-0.159 – 0.005) | 0.005 (0.003 – 0.006)***  -0.046 (-0.126 – 0.034) | --0.094 (-0.180 – -0.009)*  -0.043 (-0.129 – 0.042) | -0.086 (-0.170 – -0.001)*  -0.023 (-0.102 – 0.056) | -0.101 (-0.187 – -0.015)*  -0.083 (-0.164 – -0.002)* | -0.093 (-0.177 – -0.009)*  -0.044 (-0.123 – 0.035 | -0.093 (-0.180 – -0.006)*  -0.044 (-0.126 – 0.037) | -0.086 (-0.170 – -0.001)*  -0.023 (-0.102 – 0.056) | --0.103 (-0.189 – -0.017)*  -0.079 (-0.161 – 0.003) | 0.006 (0.004 – 0.008)***  -0.049 (-0.129 – 0.031) |
| **Sex (female)** | 0.133 (0.073 – 0.193)*** | 0.102 (0.043 – 0.161)*** | 0.129 (0.068 – 0.190)*** | 0.100 (0.041 – 0.159)*** | 0.139 (0.078 – 0.200)*** | 0.107 (0.048 – 0.166)*** | 0.131 (0.071 – 0.192)*** | 0.101 (0.042 – 0.160)*** | 0.105 (0.045 – 0.164)*** | 0.099 (0.040 – 0.158)** | 0.135 (0.075 – 0.195)*** | 0.104 (0.046 – 0.163)*** | 0.135 (0.074 – 0.195)*** | 0.099 (0.040 – 0.158)** | 0.132 (0.072 – 0.193)*** | 0.102 (0.044 – 0.161)*** |
| **BMI-SDS** | - | 0.202 (0.173 – 0.232)*** | - | 0.203 (0.174 – 0.232)*** | - | 0.209 (0.180 – 0.238)*** | - | 0.203 (0.173 – 0.232)*** | - | 0.211 (0.181 – 0.240)*** | - | 0.200 (0.171 – 0.229)*** | - | 0.211 (0.181 – 0.240)*** | - | 0.203 (0.173 – 0.232)*** |
| **TIR** | -0.005 (-0.007 – -0.003)*** | -0.004 (-0.006 – -0.002)*** | - | - | - | - | - | - | - | - | - | - | - | - | - | - |
| **TITR** | - | - | -0.005 (-0.007 – -0.003)*** | -0.004 (-0.006 – -0.002)*** | - | - | - | - | - | - | - | - | - | - | - | - |
| **CV** | - | - | - | - | 0.007 (0.002 – 0.012)** | 0.007 (0.002 – 0.012)** | - | - | - | - | - | - | - | - | - | - |
| **TAR** | - | - | - | - | - | - | 0.005 (0.003 – 0.006)*** | 0.003 (0.001 – 0.005)*** | - | - | - | - | - | - | - | - |
| **TBR** | - | - | - | - | - | - | - | - | 0.003 (-0.008 – 0.014) | 0.009 (-0.002 – 0.020) | - | - | - | - | - | - |
| **GRI** | - | - | - | - | - | - | - | - | - | - | 0.004 (0.003 – 0.005)*** | 0.003 (0.002 – 0.005)*** | - | - | - | - |
| **GRI hypo** | - | - | - | - | - | - | - | - | - | - | - | - | 0.005 (-0.008 – 0.018) | 0.011 (-0.002 – 0.024) | - | - |
| **GRI hyper** | - | - | - | - | - | - | - | - |  | - |  | - |  | - | 0.006 (0.004 – 0.008)*** | 0.004 (0.002 – 0.006)*** |
| Estimates with (95% CI)in brackets. *p<0.05; ** p<0.01; ***p<0.001. Pubertal status reference group: pre-puberty  Abbreviations: SBP, systolic blood pressure; TIR, Time in Range 70–180 mg/dL (3.9–10.0 mmol/L); TITR, Time in Tight Range 70-140 mg/dL (3.9 – 7.8 mmol/L); TBR, Time Below Range <70 mg/dL (< 3.9 mmol/L); TBR1, Time Below Range 54-69 mg/dL (3.0 - 3.9 mmol/L) (low glucose or Level 1 hypoglycaemia); TBR2, Time Below Range <54 mg/dL (<3.0 mmol/L) (very low glucose or Level 2 hypoglycaemia); TAR, Time Above range >180 mg/dL (>10.1 mmol/L); TAR1, Time Above Range 181-250 mg/dL (10.1-13.9 mmol/L) (high glucose or Level 1 hyperglycaemia); TAR2, Time Above Range >250 mg/dL (>13.9 mmol/L) (very high glucose or Level 2 hyperglycaemia); GRI, Glycemia Risk Index; CV, Coefficient of variation; SD, standard deviation. | | | | | | | | | | | | | | | | |

Supplementary table 2. Multiple linear regression models evaluating associations between CGM-derived metrics and LDL-C. Model 1: adjusted for sex and pubertal status groups; Model 2: adjusted for sex and pubertal status groups, and BMI-SDS.

| **Predictor** | **Model 1** | **Model 2** | **Model 1** | **Model 2** | **Model 1** | **Model 2** | **Model 1** | **Model 2** | **Model 1** | **Model 2** | **Model 1** | **Model 2** | **Model 1** | **Model 2** | **Model 1** | **Model 2** |
| --- | --- | --- | --- | --- | --- | --- | --- | --- | --- | --- | --- | --- | --- | --- | --- | --- |
| **Pubertal status groups**  **-puberty**  **-post-puberty** | -0.019 (-2.407 – 2.369)  -2.957 (-5.230 – -0.683)* | 0.076 (-2.301 – 2.454)  -2.602 (-4.868 – -0.335)* | -0.060 (-2.451 – 2.331)  -2.825 (-5.099 – -0.552)* | 0.048 (-2.332 – 2.429)  -2.461 (-4.728 – -0.195)* | 0.251 (-2.144 – 2.646)  -2.067 (-4.316 – 0.183) | 0.323 (-2.059 – 2.706)  -1.813 (-4.052 – 0.426) | -0.018 (-2.406 – 2.369)  -2.852 (-5.116 – -0.587)* | 0.078 (-2.300 – 2.455)  -2.506 (-4.764 – -0.248)* | -0.119 (-2.276 – 2.515)  -1.991 (-4.237 – 0.255) | 0.210 (-2.173 – 2.594)  -1.741 (-3.977 – 0.495) | 0.051 (-2.335 – 2.437)  -2.906 (-5.173 – -0.640)* | 0.135 (-2.241 – 2.510)  -2.575 (-4.834 – -0.316)* | 0.126 (-2.270 – 2.521)  -1.988 (-4.233 – 0.258) | 0.216 (-2.168 – 2.599)  -1.738 (-3.974 – 0.498) | -0.038 (-2.423 – 2.347)  -2.974 (-5.236 – -0.711)* | 0.054 (-2.321 – 2.429)  -2.638 (-4.894 – -0.381)* |
| **Sex (female)** | 8.042 (6.377 – 9.707)*** | 7.689 (6.027 – 9.352)*** | 7.960 (6.293 – 9.627)*** | 7.619 (5.955 – 9.283)*** | 8.136 (6.462 – 9.810)*** | 7.751 (6.081 – 9.421)*** | 7.998 (6.333 – 9.663)*** | 7.655 (5.993 – 9.317)*** | 7.993 (6.321 – 9.665)*** | 7.631 (5.963 – 9.299)*** | 8.099 (6.435 – 9.764)*** | 7.739 (6.077 – 9.400)*** | 7.996 (6.324 – 9.668)*** | 7.633 (5.965 – 9.301)*** | 8.027 (6.364 – 9.690)*** | 7.682 (6.021 – 9.342)*** |
| **BMI-SDS** | - | 2.336 (1.511 – 3.161)*** | - | 2.348 (1.522 – 3.174)*** | - | 2.526 (1.705 – 3.348)*** | - | 2.312 (1.486 – 3.138)*** * | - | 7.631 (5.963 – 9.299)*** | - | 2.349 (1.526 – 3.173)*** |  | 2.492 (1.669 – 3.316)*** | - | 2.296 (1.472 – 3.120)*** |
| **TIR** | -0.125 (-0.176 – -0.074)*** | -0.110 (-0.161 – -0.059)*** | - | - | - | - | - | - | - | - | - |  |  | - | - | - |
| **TITR** | - | - | -0.125 (-0.182 – -0.068)*** | -0.106 (-0.163 – -0.048)*** | - | - | - | - | - | - | - |  |  | - | - | - |
| **CV** | - | - | - | - | 0.096 (-0.047 – 0.238) | 0.095 (-0.049 – 0.239) | - | - | - | - | - |  |  | - | - | - |
| **TAR** | - | - | - | - | - | - | 0.125 (0.076 – 0.175)*** | 0.109 (0.059 – 0.158)*** | - | - | - |  |  | - | - | - |
| **TBR** | - | - | - | - | - | - | - | - | -0.271 (-0.575 – 0.034) | -0.209 (-0.513 – 0.095) | - |  |  | - | - | - |
| **GRI** | - | - | - | - | - | - | - | - | - | - | 0.101 (0.062 – 0.140)*** | 0.090 (0.051 – 0.129)*** |  | - | - | - |
| **GRI hypo** | - | - | - | - | - | - | - | - | - | - | - | - | -0.312 (-0.678 – 0.054) | -0.239 (-0.604 – 0.125) | - | - |
| **GRI hyper** | - | - |  | - | - | - | - | - | - | - | - | - | - |  | 0.169 (0.110 – 0.229)*** | 0.151 (0.092 – 0.210)*** |
| Estimates with (95% CI) in brackets. *p<0.05; ** p<0.01; ***p<0.001. Pubertal status reference group: pre-puberty.  Abbreviations:LDL-C: low-density lipoprotein-cholesterol; TIR, Time in Range 70–180 mg/dL (3.9–10.0 mmol/L); TITR, Time in Tight Range 70-140 mg/dL (3.9 – 7.8 mmol/L); TBR, Time Below Range <70 mg/dL (< 3.9 mmol/L); TBR1, Time Below Range 54-69 mg/dL (3.0 - 3.9 mmol/L) (low glucose or Level 1 hypoglycaemia); TBR2, Time Below Range <54 mg/dL (<3.0 mmol/L) (very low glucose or Level 2 hypoglycaemia); TAR, Time Above range >180 mg/dL (>10.1 mmol/L); TAR1, Time Above Range 181-250 mg/dL (10.1-13.9 mmol/L) (high glucose or Level 1 hyperglycaemia); TAR2, Time Above Range >250 mg/dL (>13.9 mmol/L) (very high glucose or Level 2 hyperglycaemia); GRI, Glycemia Risk Index; CV, Coefficient of variation; SD, standard deviation. | | | | | | | | | | | | | | | | |
